# Positive childbirth experience: A qualitative study

**DOI:** 10.1002/nop2.499

**Published:** 2020-04-14

**Authors:** Monirolsadate Hosseini Tabaghdehi, Afsaneh Keramat, Sakineh Kolahdozan, Zohreh Shahhosseini, Mahmood Moosazadeh, Zahra Motaghi

**Affiliations:** ^1^ Student Research Committee School of Nursing and Midwifery Shahroud University of Medical Sciences Shahroud Iran; ^2^ Reproductive Studies and Women's Health Research Center Shahroud University of Medical Sciences Shahroud Iran; ^3^ Department of Medicine School of Medicine Shahroud University of Medical Sciences Shahroud Iran; ^4^ Sexual and Reproductive Health Research Center Mazandaran University of Medical Sciences Sari Iran; ^5^ Health Sciences Research Center Addiction Institute Mazandaran University of Medical Sciences Sari Iran

**Keywords:** childbirth, experience, midwifery, nurses, nursing, qualitative, research

## Abstract

**Aim:**

This study aimed to explore the meaning of a positive childbirth experience expressed by women who had given birth in Iran.

**Design:**

Qualitative exploratory study.

**Methods:**

This is a qualitative study conducted on 10 women aged 20–38 years with positive childbirth experience. Data were collected using semi‐structured interviews from 72 hr–2 months after childbirth.

**Results:**

Data analysis led to into two themes and five subthemes. The themes include control and empowerment. Control theme consisted of three subthemes of preparation, coping and support; and empowerment theme consisted of two subthemes of self‐efficacy and self‐esteem. Women's sense of empowerment to childbirth can be the result of a positive childbirth experience. Therefore, it seems that providing positive experience factors of childbirth plays an important role in women's self‐efficacy and self‐esteem, which requires cooperation and effort at the level of the individual, family, education system and healthcare system.

## 1 INTRODUCTION

Women gain essential experiences during the labour process that remain with them throughout their lives. The quality of these experiences affects the health of the mother and her child, the mother–child relationship, as well as the spouse (Bayrami, Valizadeh, & Zaheri, 2011).

The experience of childbirth is multifaceted, so it is difficult to describe and explain. Various variables have been involved in evaluations of childbirth experiences, including midwife support, duration of labour, pain, expectations of labour, involvement and participation of labour, use of invasive methods such as episiotomies, forceps and emergency caesarean section (Hildingsson, Johansson, Karlström, & Fenwick, 2013).

A systematic review study by Hosseini et al. have shown that the prevalence of childbirth negative experiences varies across communities and is influenced by various factors including individual factors (age, parity, participation, control, expectations, preparation, fear, self‐efficacy), interpersonal factors (care provider support, husband support) and unexpected medical problems for mother and child (Hosseini Tabaghdehi et al., 2019).

Consequences of a positive experience of childbirth include increasing self‐esteem, self‐efficacy, skills, maternal and infant attachment and better acceptance of the maternal role (Ekström & Nissen, 2006; Goodman, Mackey, & Tavakoli, 2004).

The negative experience of childbirth leads to the choice of caesarean section or abortion for subsequent pregnancy. It also plays a role in fertility and pregnancy intervals so that negative experience will reduce fertility and increase the distance to the next pregnancy (Gottvall & Waldenström, 2002).

Studies show that fear of childbirth leads to reduce self‐efficacy and increase negative experience of childbirth. It also makes to choose caesarean for the next delivery (Al Ahmar & Tarraf, 2014; Christiaens & Bracke, 2007). Melender reported one of the reasons for fear of childbirth is to heard negative stories told by others (Hanna‐Leena Melender, 2002).

Sharing women's quotes from different societies makes childbirth as an important event in women's lives, as well as an opportunity to discuss fears, worries, feelings of hopelessness, inadequacy and recognition of women's resources. The service provider should provide an opportunity to share women's quotes from positive childbirth experiences (Callister, 2004).

Some studies of childbirth experiences have been conducted in different countries. The findings of a qualitative study in Sweden has shown that several factors affecting positive childbirth experiences including trusting their body on how deal with delivery pain, mind–body interaction and health care provider and spouse support (Nilsson, Thorsell, Hertfelt Wahn, & Ekström, 2013).

A study in Uganda found that healthcare provider support and care effects on childbirth experiences. Physical and psychology support lead to positive experience, and inappropriate communication and care lead to negative experience (Namujju et al., 2018).

In another study by Attanasio et al. in the United States, it was found that appropriate communication between women and healthcare provider during birth had a positive effect on the experience of childbirth (Attanasio, McPherson, & Kozhimannil, 2014).

Since there has been no study on the positive experience of Iranian women, the aim of this study was to explore the themes related to perception positive childbirth experience to provide useful information to healthcare providers and policymakers that promote positive childbirth experiences.

## 2 METHODS

### 2.1 Aim and design

This is a qualitative study with a content analysis method conducted on ten women aged 20–38 years with positive childbirth experience. The criteria included in the study were women with uncomplicated vaginal delivery, cephalic presentation and had healthy infants. To achieve the maximum variation, women with different parity, age and different education were selected. Data were collected during May to June 2018 in Abbas Abad Health Center of Mazandaran University of Medical Sciences, Iran. The sampling process was purposeful. Face‐ to‐face semi‐structured interviews were conducted from 72 hr–2 months after childbirth in privacy room at Abbas Abad Health Center. The interviews lasted approximately 35–60 min. In the present study, after ten interviews, the data were saturated and no new data were obtained. At first, general questions were asked to begin the interview and were guided by the participants' responses to the interview process. Initial questions included: “How did your perception childbirth?” and “What abilities did you get with natural childbirth?”

The interviews were conducted by the author in Persian and then were translated into English.

Ethical approval was given by the Research Council and the Ethics Committee of Shahroud University of Medical Sciences, and permission was also obtained from Mazandaran University of Medical Sciences. Participating in the study was voluntary for the women. Before the time interview, researcher explained purpose of the study, the confidentiality of their responses and also mentioned that there is the possibility of withdrawing from the study at any time. Then, informed written consent for participation in the research along with permission to record the interviews was obtained.

The process of data analysis was also conducted based on content analysis described by Graneheim and Lundman (2004). In the first step, the interviews were recorded by a tape recorder and then were typed on paper word‐by‐word by author, and the transcripts were reviewed for several times to obtain a general sense of understanding. In the second step, words and sentences containing information about the research question were considered as semantic units. In the third step, the meaningful units were abstracted and labelled with codes. In the fourth step, the codes were also compared with each other in terms of similarities and differences and grouped into the categories. In the final step, determining the themes based on the categories in the research team.

Some factors including long‐term engagement, insight into data collection, review by the supervisor and continuous comparison of data were used to validate the data. Dependency indicates the consistency and reliability of the data. For this purpose, additional comments from colleagues and handwriting review were used by participants. The ability to transplant the findings was determined by reporting into two experts and obtaining the same result. The transferability of the study was provided by rich data descriptions (Chiovitti & Piran, 2003; Polit & Beck, 2008).

## 3 RESULTS

Data analysis led to into two themes and five subthemes. The themes include control and empowerment. Control theme consisted of three subthemes of preparation, coping and support; and empowerment theme consisted of two subthemes of self‐efficacy and self‐esteem.

The characteristics of selected participants are mentioned in Table 1.

**TABLE 1:** The demographic characteristics of the participants

Variable	*N* (%)
Age	
20–30	7 (70)
31–40	3 (30)
Total	10 (100)
Education	
Under diploma	1 (10)
Diploma	4 (40)
University	5 (50)
Total	10 (100)
Parity	
Primipara	4 (40)
Multipara	6 (60)
Total	10 (100)
Occupation	
Housewife	8 (80)
Employed	2 (20)
Total	10 (100)

### 3.1 Control

In this study, it was found that internal and external control of labour process plays an effective role in pleasant perception of labour. Women's preparation to deal with childbirth as well as applying different approaches for coping childbirth are internal control factors and childbirth support by spouse, relatives and healthcare providers are external factors of birth control.

#### 3.1.1 Preparation

Participants in this study stated that preparation (mentally and physically) plays a significant role in the experience of childbirth. This preparation is created by knowing about the process of childbirth, planned pregnancy and delivery and familiarity with the environment. Participants in the study also stated that they received information from a variety of sources including the media, childbirth preparation classes, parental and other important items:

I liked the vaginal delivery and I was searching the internet a lot, I felt I could have a natural delivery…I also got information related to the delivery of the midwife. (p 2)

My mother always told me I had four natural births. How can you not? These words came to my mind at the time of childbirth and I could handle my birth better. (p4)

Previous familiarity with the delivery environment plays a vital role in the satisfaction of childbirth. Familiarizing women with the delivery environment before the childbirth creates realistic expectations.

The women in this study stated that:

The delivery environment was very important to me. I had gone to the delivery room before I gave birth and I imagined the space in my mind. I did not expect that much. (p8)

Some participants stated that pregnancy and childbirth should be planned to be enjoyable:

The baby is like a guest that is coming into your life but is always with you, so before you invite him/her, you have to prepare the field plan and support when you get him/her. (p5)

#### 3.1.2 Coping

In this study, it was found that women use different approaches to deal with childbirth. Participants stated that they could better deal with labour pain using cognitive approaches including distraction and beliefs as well as behavioural techniques such as deep breathing, walking and exercise. Because coping strategies affect one's perceptions of the severity of labour pain and labour control:

During labour and delivery, I always thought that God had given women the power and ability to give birth, So I can have a natural childbirth, like my mother, like my sister. (p1)

I took a deep breath, walked and did some of the exercises we were taught in the preparatory classes in order my cervix to be opened faster. (p10)

#### 3.1.3 Support

In this study, most participants stated that when they were considered, they felt safe and could better control childbirth. This attention includes care provider, spouse and relative support. Appropriate communication healthcare provider, understanding women's needs, informing women about the progress of childbirth and adequate physical care provide security and help them to have better control of delivery. In this study, participants stated that:

The midwives were working; I would ask them what my situation was right now. I wanted to inform me. Well, knowing what stage you are in and how much you have left, are very helpful. (p5)

When my pain started, I went to the hospital with my husband. I was admitted. Both the staff were good and the atmosphere was calming. The midwives listened to a baby's heart several times in one hour, I thought to myself that they work really well. (p4)

In this study, the participants also stated that the support and presence of husband and relatives play a useful role in their childbirth experience:

I wanted my spouse to be by my side during childbirth. The lonely delivery room is annoying. My husband supported me during all the pregnancy moments; he was with me at delivery and after it. (p7)

### 3.2 Empowerment

The study found that women with positive childbirth experiences increased their empowerment and became awareness of their strengths and capacities as well as their ability to cope with other life challenges**.**

#### 3.2.1 Self‐esteem

In this study, women with positive childbirth experiences find out their ability to cope, their responsibility to their family and their independence that these indicates an increase in their empowerment in the life.

Participants stated that:

When I gave birth, I was proud of myself. With childbirth your duty becomes heavier, you take more responsibility, you will be responsible for someone else. I wanted to do everything myself. (p9)

Previously, when I wanted to do something, I asked ten people; but now, I am empowered with childbirth; I think I should keep somebody else. (p2)

#### 3.2.2 Self‐efficacy

The study found that a positive experience of childbirth leads to increased self‐efficacy in women. Since self‐efficacy is an important prerequisite for behaviour in the situation, with increasing self‐efficacy, women in this study had a better understanding of the material role and were more likely to choose vaginal delivery in subsequent childbirth.

Participants stated that:

Natural childbirth is really good, you do not understand what it means to be a mother until you have the labour pain…A woman giving birth is an artist. I always wanted to have this art. (p3)

Giving birth is very sweet, there is no pain as sweet as labor pain and childbirth and I choose vaginal delivery again, if I want to give birth. (p4)

## 4 DISCUSSION

In this study, we found that controlling factors of childbirth plays an essential role in childbirth satisfaction. These include preparation, coping and support. This study showed that after a pleasant childbirth, women's self‐efficacy and self‐esteem were increased, and thus, their ability to improve their health was increased too.

Mental preparation is created by familiarity of the delivery environment and awareness of childbirth and pregnancy are planned. Physical preparation is created through relaxation training and various practical techniques in childbirth preparation classes; in fact, these methods create realistic expectations for women. Realistic expectations lead to a positive attitude towards childbirth. The result of the Dahlberg et al. study showed the effective factors of realistic expectations including books, magazines, the Internet and television, receiving information from a care provider, friends and colleagues (Dahlberg et al., 2016).

Some studies have found that modelling mothers is important in creating realistic expectations; according to Bandura's social learning, experiences are transferred from mother to child (Bandura, 1978). Health policymakers should design programmes to provide maternal and physical preparation.

Participants stated that they used different cognitive and practical approaches to cope and control to childbirth. This is supported by findings from a qualitative study (Aune et al., 2015; Bayrami et al., 2011; Karlström, Nystedt, & Hildingsson, 2015; Shahoei et al., 2014; Vaziri, Khademian, & Behbahani, 2012). Therefore, the provider must identify women's resources during pregnancy to deal with childbirth and strengthen these resources.

The result showed that the mothers who received adequate support of healthcare provider and spouse had positive childbirth experience. This result confirm by other studies (Askari et al., 2010; Aune et al., 2015; Bayrami et al., 2011; Gibbins & Thomson, 2001; Henriksen, Grimsrud, Schei, & Lukasse, 2017; Karlström et al., 2015; Nilsson et al., 2013). Healthcare provider support includes physical and psychological support such as appropriate care and communication (Ford, Ayers, & Wright, 2009). Due to the importance of communication in understanding pleasant delivery, healthcare provider must acquire enough scientific and practical skills in the field of health care.

Women stated that the presence of their husband and other companion during labour helped them better deal with the birth and several studies have suggested the role of spouse support in understanding the experience of childbirth (Gibbins & Thomson, 2001; Henriksen et al., 2017; Karlström et al., 2015; Lundgren, 2005; Nilsson et al., 2013). Healthcare providers should consult with pregnant women to identify a person who is involved in the delivery process.

Empowerment is a process through which people will have more control over decisions, lifestyles and activities that affect their health.

Self‐efficacy and self‐esteem are one of the dimensions of empowerment. One of the important implications of this study is to increase self‐efficacy. Self‐efficacy is one's belief in one's ability to perform a particular behaviour successfully, which can influence one's behaviour, thinking pattern and reaction (Bandura, 1997). Women with high self‐efficacy respond well to critical situations and events and solve problems effectively.

High self‐efficacy promotes social health including the ability to cope with social challenges, the ability to manage interpersonal disadvantages and the ability to establish and maintain interpersonal relationships and promote emotional health, including the belief in the ability to control and manage stress.

Another necessary consequence of a pleasant perception of childbirth is self‐esteem. The findings of this study are in line with a study by Shahoei et al. that shows perception of experience of childbirth positively increases patience, responsibility, self‐esteem, self‐efficacy, independence, better mother–infant relationship, promotion quality of life and women's empowerment (Shahoei et al., 2014). Women's empowerment makes women more involved in taking care of themselves and their baby, better controlling and managing other life challenges.

## 5 CONCLUSION

Women's sense of empowerment to childbirth can be the result of a positive childbirth experience that is influenced by the preparation, coping and support of the service provider and spouse. Therefore, positive experience factors of childbirth seem to play a role in women's self‐efficacy and self‐esteem, which requires cooperation and effort at the level of the individual, the family, the education system and the healthcare delivery system.

## 6 LIMITATIONS

As this study was qualitative, the obtained results cannot be generalized to the entire women population. Lack of sufficient motivation among some of the participants to interview was one of the limitations of this study, and also, this study was done in a small town without ethnic diversity.

## CONFLICT OF INTEREST

The authors have no conflicts of interest relevant to this article**.**

## AUTHORS' CONTRIBUTIONS

MHT, AK and *ZM: *Conceptualization of the study, coordination, acquisition of data and drafting of the manuscript. ZS, SK and MM: Acquisition of data and drafting of the manuscript. All authors read and approved the final manuscript.
